# T-regulatory cells in severe atopic dermatitis: alterations related to cytokines and other lymphocyte subpopulations

**DOI:** 10.1007/s00403-012-1290-9

**Published:** 2012-09-12

**Authors:** Zbigniew Samochocki, Marek Alifier, Paweł Bodera, Renata Jeziorkowska, Ewa Rosiak, Beata Jurkiewicz, Olga Glińska, Wiesław Gliński, Wanda Stankiewicz

**Affiliations:** 1 Department of Dermatology, Medical University of Warsaw, ul. Koszykowa 82A, 02-008 Warsaw, Poland; 2 Department of Clinical Immunology, Medical University in Bialystok, ul. Waszyngtona 17, 17-274 Bialystok, Poland; 3 Department of Microwave Safety, Military Institute of Hygiene and Epidemiology, ul. Szaserów 128, 04-141 Warsaw, Poland

**Keywords:** Atopic dermatitis, Immunological markers, SCORAD index, T-regulatory cells

## Abstract

The changes in lymphocyte subpopulations in atopic dermatitis (AD) concern also T-regulatory cells. We investigated the expression of various surface receptors on CD3^+^CD4^+^CD25^high^FoxP3^+^ T-regulatory cells and the activation CD28^+^ receptor and the inhibitory CD152^+^ receptor on helper/inducer as well as cytotoxic/suppressor T cells. Peripheral blood lymphocytes of 15 AD patients and 20 healthy subjects were analyzed by flow cytometry using monoclonal antibodies. The concentrations of IL-6, IL-10 and TGF-β were determined in the serum and the supernatant of ConA-stimulated CD4^+^ lymphocytes. In AD patients the percentage of CD4^+^CD25^high^FoxP3^+^ as well as CD3^+^CD8^+^ cells increased, which positively correlated with SCORAD index (*r* = 0.55, *p* = 0.03). The concentrations of IL-10 in the CD4^+^ lymphocyte culture supernatants and the concentrations of TGF-β in the sera and the supernatant negatively correlated with the severity of AD (*p* < 0.01, *r* = −0.63; *p* < 0.02, *r* = −0.64 and *p* < 0.03, *r* = −0.58, respectively), whereas the serum concentration of IL-6 correlated positively (*p* < 0.003, *r* = 0.71). The regulatory cells expressed more CD62L and CD134 surface markers but less CD95. Reduced expression of the apoptotic CD95 receptor suggests that survival time of these cells is prolonged. Since CD62L and CD134 were upregulated, the enhanced modulatory effect of CD4^+^CD25^high^FoxP3^+^ cells seemed to be suggested, which may result in increased co-expression of CD28/CD152 on both CD4^+^ and CD8^+^ subpopulations.

## Introduction

The activation of Tregs [2, 16, 17, 23] is one of the possible mechanisms of down-regulation of the inflammatory process in the skin allergic reaction. Tregs account for 5–10 % of the peripheral blood CD4^+^ cells showing the constitutive co-expression of CD25 receptor. Tregs include two main subpopulations: natural Tregs (nTregs) expressing the nuclear transcription factor–forkhead winged helix P3 (FoxP3) and inducible Tregs [13]. The nTregs are generated in the thymus. Due to the restriction of HLA they become tolerant to own antigens inducing suppression of auto-reactive T-cell clones. The population of inducible Tregs differentiates from nTregs after the stimulation by cytokines, i.e., TGF-β and IL-10 induce Tregs, IL-10 induces T-regulatory type 1 cells (Tr1) and TGF-β induces Th3/Th2 cells.

Apart from CD25 and FoxP3 receptors expressions, Tregs may express many other receptors: the cytotoxic T lymphocyte-associated protein 4 (CTLA4 = CD152), the glucocorticoid-induced tumor necrosis factor receptor-related protein (GITR = TNFRSF18), L-selectin (CD62L = 13–162L = SELL) and OX40 (CD134 = TNFRSF4). Due to different expressions of these receptors, Tregs modulate the function of other cells not only in the peripheral blood but also in the skin.

By secreting IL-10 and/or TGF-β, different types of Tregs may directly or indirectly regulate differentiation, survival and activity of Th1, Th2, mast cells, eosinophils, keratinocytes, and affect the isotype of antibodies synthesized by B cells [21].

The aim of our paper was to study (1) the numbers of T-regulatory, CD3^+^CD4^+^and CD3^+^CD8^+^cells in the peripheral blood of AD patients depending on their disease activity (2) the some receptors on these cells and (3) the concentrations of IL-6, IL-10 and TGF-β in serum and production of these cytokines by CD4^+^ lymphocyte cultures.

## Materials and methods

The study included 15 AD patients: 5 females, 10 males, aged between 18 and 43, mean 27.9 years old with active skin lesions; and 20 healthy non-atopic age- and sex-matched volunteers without any personal/family history and clinical atopy symptoms. The mean SCORAD index [4] was 60.1 (range 37.9–86.3) and the mean total IgE level was 2,532 IU/ml (range 134–9,770) in the AD group. All AD patients fulfilled the diagnostic criteria of Hanifin and Rajka [6]. Six out of 15 AD patients had seasonal allergic rhinitis and three showed seasonal atopic asthma symptoms. Only routine treatment of AD including emollients, topical corticosteroids and oral anti-histamines was accepted. AD patients with concomitant asthma were allowed to take inhaled steroids. The study was approved by the Local Bioethics Committee.

### Flow cytometry

**Fig. 1:**
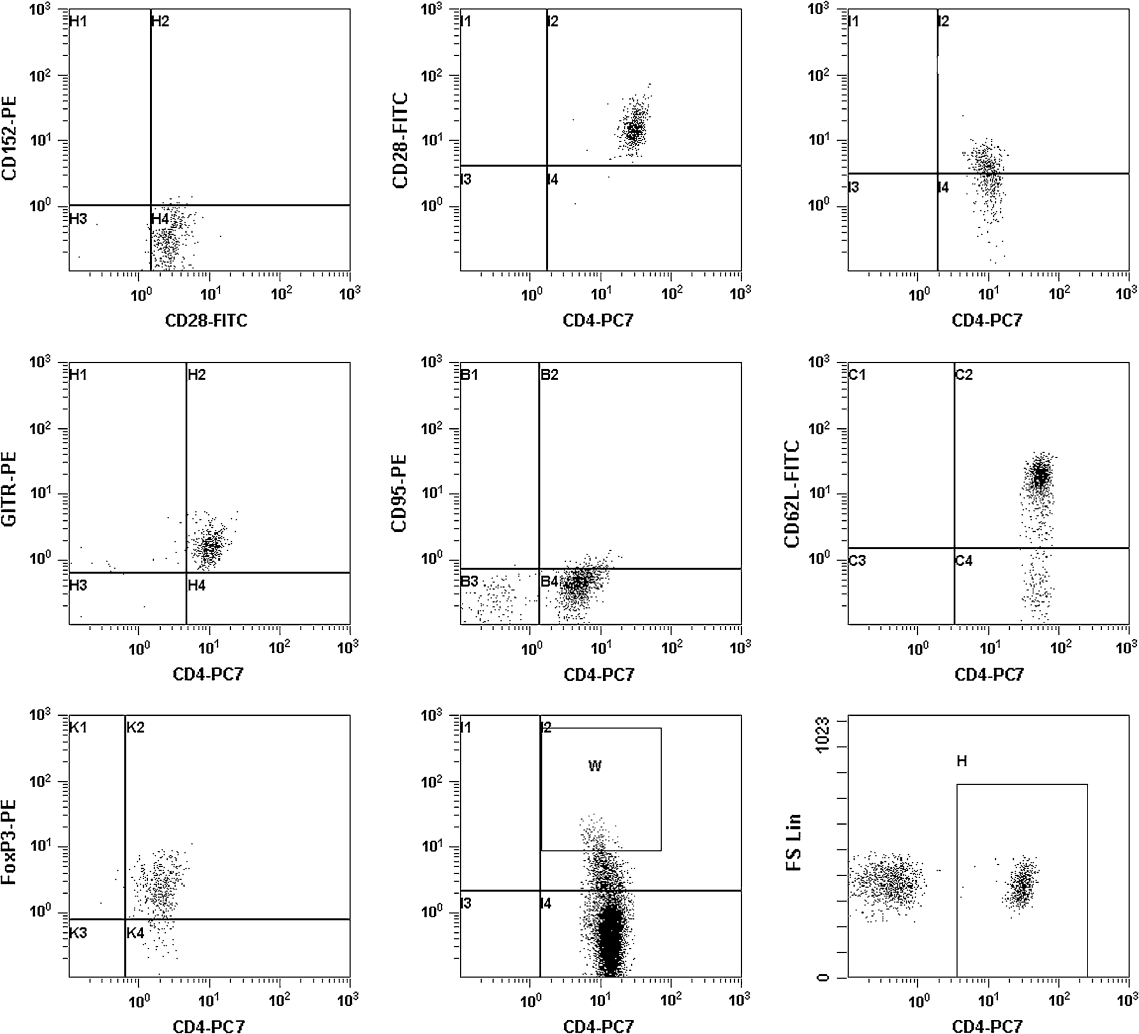
The gating of cells in flow cytometry studies. **a** Gate H, CD4^+^ lymphocytes; **b** gate W, CD4^+^CD25^high^ lymphocytes; **c** gate K2, CD4^+^CD25^high^FoxP3^+^ lymphocytes; **d** gate C2, CD4^+^CD25^high^CD62L lymphocytes; **e** gate B2, CD4^+^CD25^high^CD95^+^ lymphocytes; **f** gate H2, CD4^+^CD25^high^GITR^+^ lymphocytes; **g** gate I2, CD4^+^CD25^high^CD134^+^ lymphocytes; **h** gate I2, CD4^+^CD28^+^ lymphocytes; **i** gate H1, CD4^+^CD152^+^ lymphocytes; gate H4, CD4^+^CD28^+^ lymphocytes; gate H2, CD4^+^CD28^+^CD152^+^ lymphocytes

Mononuclear cells were isolated from peripheral blood by centrifugation over Histopaque (Sigma). A flow cytometric analysis of T-cell subpopulations was performed using the following markers: anti-CD3 (phycoerythrin-cyanin 5 PECy5 conjugated, UCHT1 clone), anti-CD4 (phycoerythrin-cyanin 7 PECy7 conjugated, SFCI12T4D11 clone), anti-CD8 (phycoerythrin-cyanin 5 PECy5 conjugated, B9.11 clone), anti-CD25 (phycoerythrin-Texas Red ECD conjugated, B1.49.9 clone), anti-CD69 (phycoerythrin conjugated, TP1.55.3 clone), anti-CD62L (fluorescein isothiocyanate conjugated, DREG56 clone), anti-hGITR (phycoerythrin conjugated clone 109101), anti-CD134 (phycoerythrin conjugated, Ber-ACT35 clone), anti-CD95 (phycoerythrin conjugated, 7C11 clone), anti-CD28 (fluorescein isothiocyanate, CD28.2 clone), anti-CD152 (phycoerythrin conjugated, BMI3 clone) and FoxP3 (phycoerythrin PE conjugated, 259D/C7 clone) purchased from Beckman Coulter (Brea, CA, USA), Beckton Dickinson (San Jose, CA, USA) and eBioscience (San Diego, CA, USA). Respective isotype control antibodies were used. Intracellular staining was performed according to the manufacturer’s instructions (Fix/Perm buffer from Beckton Dickinson). The samples were analyzed by five-colour flow cytometer Beckman Cytomics FC 500 using CXP software ver 2.0 (Beckman Coulter). A minimum of 10^5^ events were acquired for each analysis. The percentages of positive cells were calculated. To determine absolute cell counts, a small volume of blood was analyzed for complete blood count (CBC) with differential mode. The absolute counts were determined by multiplying the frequency of positive cells obtained in cytometric analysis by the number of lymphocytes [G/L] as determined by CBC. The following subpopulations were noted: CD4^+^, CD4^+^CD25^high^, CD4^+^CD25^high^FoxP3^+^, CD4^+^CD25^high^CD62L^+^, CD4^+^CD25^high^CD134^+^, CD4^+^CD25^high^CD95^+^, CD4^+^CD25^high^GITR^+^ CD4^+^CD25^high^CD152^+^, CD4^+^CD28^+^, CD4^+^CD152^+^, CD8^+^CD28^+^ and CD8^+^CD152^+^. The gating of lymphocytes is presented in Fig. 1. Cells expressing CD4^+^CD25^high^FoxP3^+^ were determined as Tregs.

Peripheral blood mononuclear cells were suspended in the concentration of 10^6^ cells per 1 ml culture medium supplemented with 10 % fetal calf serum (WSS, Lublin) and were cultured in the presence of 20 μg concanavalin A/ml of culture medium. The cells were incubated for 72 h in humidified atmosphere enriched with 5 % CO_2_ (Hereus incubator). The supernatants from the cell cultures were frozen at −20 °C.

The concentrations of IL-6, IL-10 and TGF-β in the serum and the supernatant from the culture of isolated CD4^+^ lymphocytes stimulated with ConA were examined by ELISA method using commercial sets (Quantikine R&D System, USA). The results were read from the calibration curve calculated from standard sera of known cytokine concentrations in pg/ml.

Total IgE serum level was established by the ELISA method using UniCAP fluorometer (Pharmacia&Upjohn). Values above 100 IU/ml were regarded as increased.

The statistical analysis was performed using Statistica software version 9.0 (Statsoft, Poland). To compare variables between two subgroups of patients depending on the disease severity or IgE level, Mann–Whitney *U* test was used. To compare the observed mean values in the patients with that in the control group, one-sample test was performed. Pearson correlation coefficient was used to assess the correlation. In all calculations *p* < 0.05 was regarded as statistically significant.

## Results

### CD4^+^ and CD8^+^ subpopulations of T cells in AD

In AD patients the percentage of helper/inducer CD3^+^CD4^+^ subpopulation of peripheral blood lymphocytes (31.7 ± 5.2) was lower as compared to that of controls (43.7 ± 5.1, *p* < 0.001), whereas the percentage of CD3^+^CD8^+^ cytotoxic/suppressor lymphocytes (30.6 ± 10.3) did not differ as compared to controls (26.5 ± 4.9, *p* > 0.05) (Table 1). This resulted in the significantly (*p* < 0.03) decreased Th/Ts ratio in AD patients (1.03 ± 0.4) versus the control group (1.6 ± 0.6).

**Table 1:** The percentage of T cells (mean ± SD) in the peripheral blood of AD patients

Group	Subpopulation of T lymphocytes			
	CD3^+^CD4^+^	CD3^+^CD4^+^CD25^high^	CD3^+^CD4^+^CD25^high^FoxP3^+^	CD3^+^CD8^+^
All AD patients (*n* = 15)	31.7 ± 5.2c	11.3 ± 4.7b	9.2 ± 3.7b	30.6 ± 10.3
AD patients with SCORAD <60 (*n* = 7)	36.6 ± 2.5b	9.6 ± 1.5c	7.5 ± 1.2c	20.8 ± 1.6c
AD patients with SCORAD >60 (*n* = 8)	27.7 ± 2.6c*	12.7 ± 1.8c*	11. 2 ± 1.5c*	39.2 ± 5.2c*
Control (*n* = 20)	43.7 ± 5.1	3.7 ± 1.5	2.8 ± 1.2	26.5 ± 4.9

^a,b,c^Statistically different from controls: ^a^ *p* < 0.05, ^b^ *p* < 0.001, ^c^ *p* < 0.0001

* *p* < 0.01; statistical difference between the SCORAD index >60 groups versus SCORAD index <60 groups

### Relation of T cell numbers to the AD activity

Patients with SCORAD >60 had the percentage of CD3^+^CD4^+^ cells much lower than those with SCORAD <60 (*p* < 0.01) and controls (*p* < 0.0001). However, the percentage of CD3^+^CD8^+^ cells in this group of patients was increased as compared to AD patients with SCORAD <60 (*p* < 0.01) and controls (*p* < 0.0001) (Table 1). The percentage of CD3^+^CD4^+^ lymphocytes negatively correlated with the severity of AD expressed by SCORAD index (*r* = −0.82, *p* = 0.0002) in contrast to the percentage of CD3^+^CD8^+^ lymphocytes that correlated positively (*r* = 0.9, *p* = 0.0001).

### CD28^+^ and CD152^+^ on CD4^+^ and CD8^+^ cells

**Fig. 2:**
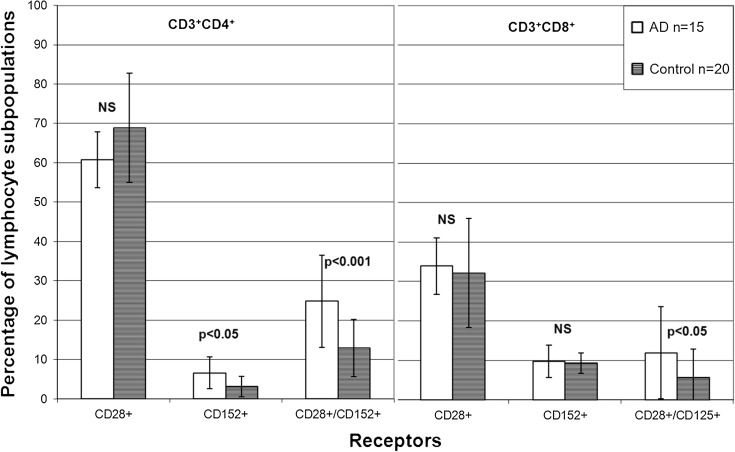
The percentage of CD3^+^CD4^+^ and CD3^+^CD8^+^ cells (mean ± SD) with the expression of CD28 and CD152 markers in AD patients

The changes in AD of the co-expression of CD28^+^ and CD152^+^ particles on both T-helper/inducer CD3^+^CD4^+^ and T-cytotoxic/suppressor lymphocytes CD3^+^CD8^+^ are presented in Fig. 2. Despite the decrease in total CD3^+^CD4^+^ cell subpopulation in AD patients, these lymphocytes showed a marked elevation of CD152^+^ expression alone (6.6 ± 4.1 %) as well as co-expression of both CD152^+^ and CD28^+^markers (24.8 ± 11.7 %) as compared to controls (3.2 ± 2.6 %, *p* < 0.05 and 12.9 ± 7.9 %, *p* < 0.001, respectively). The percentage increase of CD3^+^CD8^+^ cytotoxic/suppressor T cells bearing CD152^+^/CD28^+^ markers was found higher in AD patients than that in controls (11.9 ± 7.1 vs. 5.7 ± 3.9, *p* < 0.05, respectively).

### T-regulatory cells within CD3^+^CD4^+^ T cells

The percentage of CD3^+^CD4^+^CD25^high^ was found to be significantly elevated (11.3 ± 4.7) as compared to non-atopic volunteers (3.7 ± 1.5, *p* < 0.001). The CD3^+^CD4^+^CD25^high^ cells bearing FoxP3 receptor were highly (*p* < 0.001) increased in AD patients (9.2. ± 3.7 %) versus controls (2.8 ± 1.2 %) (Table 1).

### T-regulatory cells: relation to SCORAD

The percentages of CD4^+^CD25^high^ and CD4^+^CD25^high^FoxP3^+^ cells were markedly higher in AD patients and SCORAD >60 than in those showing SCORAD <60 (*p* < 0.01), whereas both groups showed an increase in these subpopulations of cells as compared to controls (*p* < 0.001) (Table 1).

The percentage of CD4^+^CD25^high^ lymphocytes positively correlated with the severity of AD expressed by SCORAD index (*r* = 0.55, *p* = 0.03), similar to the percentage of CD4^+^CD25^high^FoxP3^+^ cells (*r* = 0.6, *p* = 0.01).

There was no correlation between total IgE concentration in AD patients and the percentages of either CD4^+^CD25^high^ or CD4^+^CD25^high^FoxP3^+^ cells.

### Receptor expression on T-regulatory cells

**Fig. 3:**
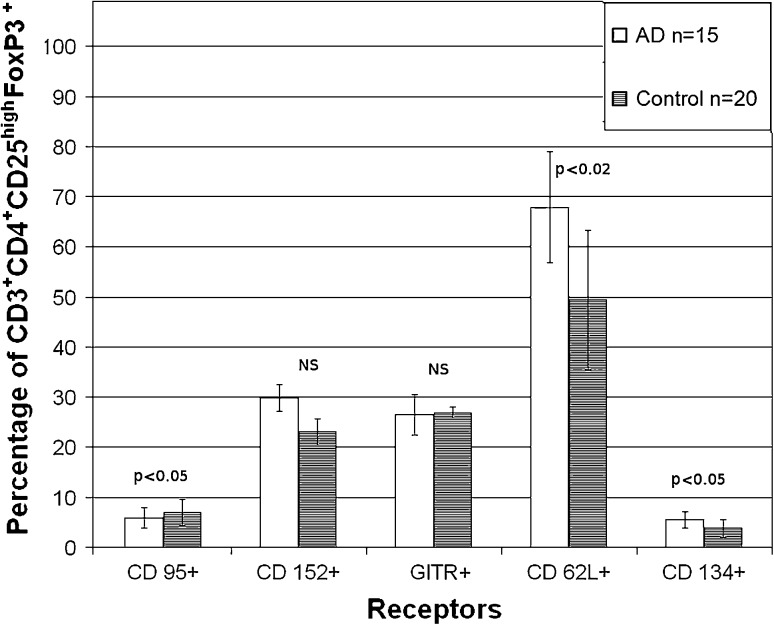
The percentage of CD3^+^CD4^+^CD25^high^FoxP3^+^ regulatory cells (mean ± SD) with the expression of various receptors in AD patients

The co-expression of various receptors on CD3^+^CD4^+^CD25^high^FoxP3^+^ regulatory cells is presented in Fig. 3. AD patients were found to have slightly lower percentage of these regulatory cells bearing CD95^+^ apoptotic receptor (5.9 ± 2.1) in comparison with controls (7.9 ± 2.6, *p* < 0.05). CD152 and GITR particles, which are responsible for the activation of suppressive function, were not altered in AD patients whereas CD62L and CD134 receptors, which are able to modulate the function of other lymphocytes, were markedly increased, reaching 67.9 ± 11.0 and 5.5 ± 1.6 %, respectively, as compared to controls (49.4 ± 13.9 %, *p* < 0.02 and 3.8 ± 1.7 %, *p* < 0.05).

### Cytokine production by lymphocyte cultures related to the disease activity

The concentration of IL-10 and TGF-β in the serum and the concentration of IL-10 in CD4^+^ lymphocyte culture supernatant were decreased in AD patients as compared to controls (17.8 ± 3.0 vs. 19.9 ± 3.6 pg/ml, *p* < 0.05; 21.9 ± 4.5 vs. 27.9 ± 5.9 pg/ml, *p* < 0.001 and 840 ± 210 vs. 1,120 ± 340 pg/ml, *p* < 0.001, respectively). The concentration of Il-6 in the serum was markedly increased (13.4 ± 6.1 vs. 7.8 ± 2.6 pg/ml, *p* < 0.01, respectively) (Table 2).

**Table 2:** The concentration of cytokines (mean ± SD, pg/ml) in serum and in culture supernatant of CD4^+^ cells in AD patients

Group	IL-10		TGF-β		IL-6	
	Serum	Supernatant	Serum	Supernatant	Serum	Supernatant
All AD patients (*n* = 15)	17.8 ± 3.0a	840 ± 210c	21.9 ± 4.5b	2510 ± 349	13.4 ± 6.1b	912 ± 271
AD patients with SCORAD <60 (*n* = 7)	17.7 ± 3.6	990 ± 77b	25.1 ± 1.1b	2696 ± 118	9.0 ± 1.5	817 ± 327
AD patients with SCORAD >60 (*n* = 8)	17.8 ± 2.6	707 ± 201c**	19.1 ± 4.3c**	2347 ± 408	17.2 ± 6.0b*	930 ± 281
Control (*n* = 20)	19.8 ± 3.6	1120 ± 340	27.9 ± 5.9	2679 ± 381	7.8 ± 2.6	845 ± 250

^a,b,c^Statistically different from controls: ^a^ *p* < 0.05, ^b^ *p* < 0.001, ^c^ *p* < 0.0001

* *p* < 0.05, ** *p* < 0.01; statistical difference between the SCORAD index >60 groups versus SCORAD index <60 groups

The patients with SCORAD >60 showed significant decrease in the concentration of IL-10 in supernatant and TGF-β in the serum as compared to the patients with SCORAD <60 (707 ± 201 vs. 990 ± 77 pg/ml, *p* < 0.01 and 19.1 ± 4.3 vs. 25.1 ± 1.1 pg/ml, *p* < 0.01, respectively) and the control group (*p* < 0.01). The concentration of IL-6 (17.2 ± 6.0 pg/ml) in the serum in patients with SCORAD >60 was significantly (*p* < 0.01) higher than in those showing SCORAD <60 (9.0 ± 1.5 pg/ml) and in controls (*p* < 0.01) (Table 2).

The concentrations of IL-10 in the CD4^+^ lymphocyte culture supernatants and the concentrations of TGF-β in the sera and the supernatant negatively correlated with the severity of AD expressed by SCORAD index (*p* < 0.01, *r* = −0.63; *p* < 0.02, *r* = −0.64 and *p* < 0.03, *r* = −0.58, respectively), whereas the serum concentration of IL-6 correlated positively (*p* < 0.003, *r* = 0.71).

## Discussion

### Regulatory T cells in the peripheral blood

The marked increase in CD3^+^CD4^+^CD25^high^FoxP3^+^ Tregs has been found in AD as compared to non-atopic controls, which is in agreement with previous reports on the increased percentage of these cells in the peripheral blood in AD [8, 10, 11, 14, 20]. In addition, the increase in both subpopulations CD4^+^CD25^high^ and CD4^+^CD25^high^FoxP3^+^ positively correlates with the activity of skin lesion expressed by SCORAD index. The inverse relationship between the increased percentage of Tregs and the diminished serum concentration and production of TGF-β and IL-10 by CD4^+^ cultured lymphocytes has been found mainly in patients with severe AD (SCORAD >60). This does not support the argument that TGF-β and IL-10 are responsible for the induction of T-regulatory cells in AD patients.

Despite FoxP3 gene transcription by TGF-β1 is vital mechanism of regulatory T cell stimulation [12], recent findings showed that FoxP3^+^ regulatory cells, of potent anti-inflammatory activity, might be subgrouped based on the chemotactic receptor expression into different Th cell subsets, each functionally suppressive [3].

Szegedi et al. [20] have not found the percentage of FoxP3^+^ Tregs to be higher than in controls, whereas these cells were reported to be markedly increased in the three other studies [8, 14, 22]. The data of Szegedi et al. [20] indicate that neither the severity of the disease nor the total IgE level influences the number of FoxP3^+^ T-regulatory cells. In our AD patients, who were subdivided by the SCORAD index as moderate and severe AD, the numbers of Tregs are elevated in both activity subgroups, whereas this phenomenon is not related to the increased total IgE level. Some authors [2] correlate the increased percentage of these cells with the severity of skin lesions. In addition, Taylor et al. [22] show that in 6-month-old infants the expression of FoxP3 receptor on CD4^+^CD25^high^ peripheral blood cells is higher in those patients who developed AD lesions later than others.

Some authors, who do not report increased number of circulating CD4^+^CD25^high^FoxP3^+^ cells, have found their accumulation in the skin lesions [5, 18]. However, this is not confirmed by Verhagen et al. [26].

### Receptors on regulatory T cells

In our study, the decreased percentage of CD95^+^ on Tregs indicates that activated CD3^+^CD4^+^CD25^high^ FoxP3^+^ cells can prolong survival, which, at least partly, contributes to their increased percentage in the peripheral blood.

The presence of surface T-regulatory receptors, i.e., CD152 (CTLA-4) and GITR that triggers the function of T-regulatory cells as well as enhanced co-expression of both CD62L and CD134 molecules, argues for their activation. It is possible that the activity of CD3^+^CD4^+^CD25^high^FoxP3^+^ cells may modulate the function of other lymphocytes more effectively.

### Cytokine production in severe AD

Both the increased production of IL-6 by cultured CD4^+^ lymphocytes and the increased serum concentration of IL-6 indicate that the effector T cells are stimulated. Such stimulation is present in the inflammatory diseases and it seems to be secondary to the disease severity and widespread skin involvement.

In our study, concentrations of both serum IL-10 and TGF-β and production of these cytokines by lymphocyte cultures have been significantly decreased, which points out to insufficient suppression of the immunological response by these cytokines in severe AD. This is consistent with the data by Antiga et al. [1], who report decreased serum levels of IL-10 and TGF-β in AD similar to the patients with lupus erythematosus. Vakirlis et al. [24] report IL-10 decrease in the patients with active phase of AD as compared to chronic AD cases and controls; however, these data are not correlated with SCORAD index. In addition, severe atopic dermatitis is associated with reduced frequency of IL-10-producing allergen-specific CD4^+^ T cells [19]. Immunosuppressive cytokines, i.e., IL-10 and TGF-β are reported to be found in AD patients, which are linked to relative prevalence of Th2 cells [9, 25, 27].

### The relationship of regulators T cells and other lymphocyte subpopulations

The T suppressor/cytotoxic CD3^+^CD8^+^ cells were significantly increased in patients with severe AD whose SCORAD was above 60. Recent studies by Hennino et al. [7] show that these cells are recruited early to allergen exposure sites and are involved in the initiation of skin inflammation in the mouse model and in humans. This indicates that increase in CD3^+^CD8^+^ T cells does not necessarily reflect the suppressive activity of this subpopulation. It seems likely that cytotoxic CD8^+^ cells are responsible for the apoptosis of keratinocytes and for the epidermal spongiosis in AD.

Our AD patients have shown the increased expression of CD152 on CD3^+^CD4^+^ cells as well as co-expression of CD28/CD152 on both CD3^+^CD4^+^ and CD3^+^CD8^+^ cell populations. The pool of double stained cells bearing both CD28 and CD152 markers may cooperate in limiting the intensity of inflammation by effector cells in skin lesions. These cells, depending on the stimulatory signal, may transform into lymphocytes expressing either activating CD28 or inhibitory CD152 receptors [15, 16].

In conclusion, the increased number of Tregs in AD has been found. These cells had upregulated CD62L and CD134 and decreased the expression of apoptotic CD95 receptor suggesting that their survival is prolonged. The immunological response pattern has been identified in the most severe AD patients. The increased percentages of both T-regulatory and T-suppressor/cytotoxic cells and the elevated serum IL-6 coexist with decreased T-helper/inducer cells as well as lower concentrations of TGF-β and IL-10 in the serum and in CD4^+^lymphocyte culture supernatants.
